# Computational Protein Design: Validation and Possible Relevance as a Tool for Homology Searching and Fold Recognition

**DOI:** 10.1371/journal.pone.0010410

**Published:** 2010-05-05

**Authors:** Marcel Schmidt am Busch, Audrey Sedano, Thomas Simonson

**Affiliations:** Laboratoire de Biochimie (CNRS UMR7654), Department of Biology, Ecole Polytechnique, Palaiseau, France; University Paris 7, France

## Abstract

**Background:**

Protein fold recognition usually relies on a statistical model of each fold; each model is constructed from an ensemble of natural sequences belonging to that fold. A complementary strategy may be to employ sequence ensembles produced by computational protein design. Designed sequences can be more diverse than natural sequences, possibly avoiding some limitations of experimental databases.

**Methodology/Principal Findings:**

We explore this strategy for four SCOP families: Small Kunitz-type inhibitors (SKIs), Interleukin-8 chemokines, PDZ domains, and large Caspase catalytic subunits, represented by 43 structures. An automated procedure is used to redesign the 43 proteins. We use the experimental backbones as fixed templates in the folded state and a molecular mechanics model to compute the interaction energies between sidechain and backbone groups. Calculations are done with the Proteins@Home volunteer computing platform. A heuristic algorithm is used to scan the sequence and conformational space, yielding 200,000–300,000 sequences per backbone template. The results confirm and generalize our earlier study of SH2 and SH3 domains. The designed sequences ressemble moderately-distant, natural homologues of the initial templates; *e.g.*, the SUPERFAMILY, profile Hidden-Markov Model library recognizes 85% of the low-energy sequences as native-like. Conversely, Position Specific Scoring Matrices derived from the sequences can be used to detect natural homologues within the SwissProt database: 60% of known PDZ domains are detected and around 90% of known SKIs and chemokines. Energy components and inter-residue correlations are analyzed and ways to improve the method are discussed.

**Conclusions/Significance:**

For some families, designed sequences can be a useful complement to experimental ones for homologue searching. However, improved tools are needed to extract more information from the designed profiles before the method can be of general use.

## Introduction

Protein sequence databases continue to grow rapidly, with 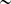
6 million entries in Uniprot [1]–[8]. Knowledge of the 3D structure is essential for understanding function; unfortunately, experimental structure determination is only practical for a small fraction of these proteins [2], [8]–[10]: just 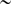
2% have an experimentally-verified structural annotation today. For the others, structure must be predicted. Thus, the structural characterization of proteins is a major goal in computational biology [1]–[8].

Structure prediction is often done on a domain basis. Indeed, most protein structures can be subdivided into one or more compact domains, which have their own independent fold. Known domain structures can be classified into a few thousand families, collected in public databases such as Pfam and SCOP [11]–[14]. To characterize the 3D structure of a new protein sequence, the first step is to identify one or more homologous proteins of known structure; from these, one can infer, or “recognize” the new protein's domains and their respective folds. The fold can be viewed as a medium resolution model of each domain's 3D structure. In a second step, the model can be refined using established homology modeling techniques [15]–[17].

Fold recognition tools [18]–[31] usually compare a new sequence to a library of virtual, consensus sequences, each representing a statistical description of one structural family. For example, the “Protein Family” or Pfam database provides a library of multiple sequence alignments (MSAs), representing 10340 distinct families of domain structures [22]–[24]. The SUPERFAMILY library is another collection of MSAs [25]–[27], which is based on the “Structural Classification of Proteins”, or SCOP database [11], [12]. SCOP currently groups the known domain structures into 3464 families. SUPERFAMILY provides one profile Hidden Markov Model (HMM) for each family, and also one for each individual SCOP domain.

Currently, fold recognition tools are able to classify (“recognize”) 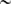
75% of the sequences in SwissProt or TrEMBL. Some of the unrecognized sequences must correspond to 3D domain structures that are as yet unknown. Indeed, new protein folds are still being discovered, albeit at a slow rate [32], [33]. Others must have a low sequence similarity to their homologues of known structures. Thus, if a structural family is not sufficiently represented in sequence databases, the MSAs and statistical models used for fold recognition may not be sufficiently representative. In general, relying entirely on experimental sequences and structures can be a limitation. Therefore, it is of interest to examine the potential of computationally-designed sequences as an aid for fold recognition [34]–[42].

Computational protein design, or CPD, represents a rigorous test of our understanding of the biophysical mechanisms that shape protein sequences and structures [35]–[39], [43]–[65]. The present implementation uses a molecular mechanics description of the protein, a simple implicit solvent model, a fixed backbone, and sidechain rotamers; the unfolded state is treated with a simple tripeptide model [42], [66]–[68]. In principle, CPD can easily generate hundreds of thousands of sequences for a single backbone template, potentially improving the exploration of sequence space in cases where experimental data is rare.

CPD's usefulness for fold recognition was considered by several groups, but has not been determined conclusively [34]–[39], [41], [42]. More generally, the value of structure-based alignments for fold recognition is still unclear [25], [40], [69]. Larson & Pande selected 253 small proteins from the Protein Data Bank. For each protein, they generated 700–800 low-energy sequences. The designed sequence profiles were used for homology searching, performing better than a single pairwise BLAST search using a natural query [35]–[37]. Zhou & Zhou used sequence profiles obtained not from CPD, but from structural alignment of small protein fragments, in combination with traditional sequence profiles. Including structure-based information at the fragment level led to excellent homology detection [40]. In a recent study, we designed sequences for 46 SH2 and SH3 domains [42]. The sequences ressembled moderately-distant homologues of the original template sequences, and the diversity within the designed sequence ensembles was comparable to that of the natural families. We then tested the designed sequences for homology searching. Position Specific Scoring Matrices (PSSMs) were derived from the designed sequences and used with the PSI-Blast search algorithm. The designed PSSMs retrieved 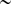
67% of the sequences found by experimental PSSMs, or 75% when explicit functional information was added by resetting a few functional positions to their native amino acid types.

Here we generalize the analysis, by considering proteins from another four SCOP families: 8 Small Kunitz-type inhibitors (SKIs), 12 Interleukin-8 chemokines, 17 PDZ domains, and 6 large Caspase catalytic subunits, for a total of 43 structures. These families come from four different structural classes in SCOP: all-beta (PDZ domains), alpha+beta (chemokines), alpha/beta (caspases), and “small proteins” (SKIs). The chemokines and PDZ domains studied correspond to 1/2 and 1/3 of their respective SCOP families. The SKIs and caspases correspond to nearly the entire SCOP families (8/8 SKIs and 6/7 caspases of known Xray structures). The PDZ family was chosen because it has been subjected to extensive analysis and redesign [70]. The SKI and caspases families were chosen because of their small size, while the chemokines are biologically interesting. All these proteins are larger than the SH2 and SH3 domains. The SKIs and chemokines each have a highly conserved disulfide bond pattern that helps preserve the stability of the fold [71]. As before, we did a basic quality control, computing similarity scores and sequence entropies, and applying several fold recognition tools. In particular, SUPERFAMILY classifies 85% of our 8,000 lowest-energy designed sequences as native-like (compared to about 82% for the SH2 and SH3 domains [42]).

We then tested the designed sequences for homology searching with PSI-Blast. For the caspases and PDZ domains, designed PSSMs retrieved about 60% of the experimental sequences in SwissProt; this is poorer than in the earlier SH2 and SH3 study [42]. For SKIs and chemokines, however, the retrieval rate was much better, around 90%. While this is still less than 100%, it does suggest that designed sequences can be a useful aid for some protein families.

A limitation of PSI-Blast as a search tool is that it employs a sequence profile, which contains less information than the original MSA used to create it. Indeed, the profile is obtained by averaging each column of the MSA, yielding a set of amino acid frequencies for each column [18]–[30]. This averaging destroys information on correlated mutations, where two positions in the polypeptide chain mutate at the same time. To better understand the correlations, we consider sequences designed under special restrictions; for example, without taking into account inter-sidechain correlations. We also analyze the contribution of different energy terms (steric packing, electrostatics, solvation) to the sidechain interactions and the overall stability of the designed proteins. Finally, for one protein, we present data on the correlated mutations and their effect on homologue searching. This analysis should help point the way to methods that extract more information from the designed sequences and give improved performance for homologue retrieval. The designed sequences are available at http://biology.polytechnique.fr/biocomputing/sequences.

## Materials and Methods

The CPD implementation was described in detail recently [42], [67]. Here, we summarize it more briefly.

### Folded and unfolded states

In the folded state, the backbone is kept fixed, while sidechains occupy standard rotamers [72]. The backbone conformation was obtained by subjecting the crystal structure to 500 steps of conjugate gradient energy minimization, with a uniform dielectric constant of 20 applied to the Coulomb electrostatic energy term. This typically led to an rms deviation (backbone and C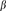 atoms) of 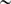
0.7 Å from the crystal structure. In the unfolded state, the amino acid sidechains do not interact with each other, but only with nearby backbone and with solvent. Specifically, for each amino acid type X, we considered a large number of possible tripeptide structures with the sequence Ala-X-Ala. The lowest-energy combination of backbone structure and sidechain rotamer was taken to represent the preferred structure of X in the unfolded state. The corresponding energy, 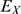, represents the contribution of X to the unfolded state free energy. An additional (and smaller) contribution, 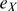, was determined empirically, so as to obtain accurate overall amino acid compositions in the final computed sequences; more details are given elsewhere [67], [68].

### Effective energy function

The effective energy function was described in detail elsewhere [66]. Briefly, we use the Charmm19 molecular mechanics energy function [73] along with the CASA implicit solvent model. With CASA, the solvent contribution is the sum of a screened Coulomb term and a solvent accessible surface term:
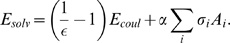
(1)Here, 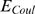 is the usual Coulomb energy, 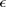 is a dielectric constant, equal to ten; the righthand sum is over the protein atoms 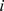, 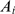 is the solvent accessible surface area of atom 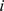, 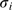 is an atomic solvation coefficient which depends on the atom type, and 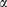 is an overall scaling factor for the surface term.

The interaction energy between each pair of sidechains, or between a sidechain and the backbone, involved a short energy minimization stage [50]. Each sidechain was first subjected to 15 steps of Powell minimization, with the backbone fixed and inter-sidechain interactions excluded. Then, interactions between the sidechain pair were included and a further 15 steps of minimization performed. The sidechain interaction energy was taken from this last, minimized structure. Interactions between distant groups were omitted through a cutoff scheme [67].

Surface areas were computed using the Lee and Richards algorithm [74], using a 1.4 Å probe radius. The atomic solvation coefficients 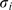 are the ones used in our previous work: 0.012 kcal/mol/Å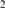 for carbons and sulfur; −0.06 kcal/mol/Å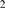 for oxygen and nitrogen; zero for hydrogens, and −0.15 kcal/mol/Å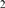 for ionized groups [66]. For reasons of efficiency, following Street & Mayo [75], we assume that 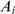 can be obtained by summing the contact areas 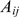 between atom 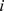 and its neighbors 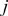, and subtracting the contact, or solvent-inaccessible area 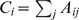 from the total area of atom 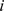. This approximation has the enormous advantage that the surface energy takes the form of a sum over pairs of amino acids [66], [75].

### Sequence optimization

We used a heuristic procedure developed by Wernisch et al. [50], [67]. A “heuristic cycle” proceeds as follows: an initial amino acid sequence and set of sidechain rotamers are chosen randomly. They are improved in a stepwise way. At a given amino acid position 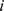, the best amino acid type and rotamer are selected, with the rest of the sequence held fixed. The “best” choice is defined as the one that maximizes the protein folding free energy. The same is done for the following position 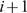, and so on, performing multiple passes over the amino acid sequence until the energy no longer improves (or a set, large number of passes is reached). The final sequence, rotamers, and energy are output, ending the cycle. For the design calculations below, we performed 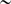
300.000 heuristic cycles. Cysteines, glycines, and prolines are expected to have a special effect on the protein's folded and unfolded state structures, which may not be accurately captured by our method. Therefore, if these amino acids are present in the native sequence, they are not mutated; all other amino acids are allowed to mutate freely (but not into Cys, Gly, or Pro).

### Software implementation

The pairwise energy function and discrete conformational space imply that all the relevant energy data can be precomputed and stored [50]. In effect, we must compute the interactions between all pairs of amino acids in the structure, allowing for all possible pairwise combinations of amino acid types and rotamer values. This calculation is done with the XPLOR program [76]. Because of its low communication requirements, the calculation can be done in parallel. We employed our Proteins@Home distributed computing platform, which allows us to use the computers of several thousand volunteers in over 100 countries (see the list of participants at biology.polytechnique.fr/proteinsathome). Proteins@Home is based on the Berkeley Open Infrastructure for Network Computing, BOINC [77].

### Similarity scores

To measure the quality of the designed sequences, we computed similarity scores between each designed sequence and a multiple sequence alignment (MSA) of experimental sequences. We used the Pfam alignments, which include, respectively: 154 SKIs, 112 chemokines, 70 PDZ domains, and 124 Caspases. To each Pfam MSA, we added the proteins studied here (the native sequences, not the designed sequences). For a given family, each of the proteins studied here was aligned separately to the original Pfam alignment (unless it was already part of that alignment). The final alignment, including the original Pfam set plus our own, additional proteins, will be referred to as the Pfam MSA, even though it is enlarged by a few additional proteins. For the Chemokines, PDZ domains, and Caspases we also calculated the similarity scores from a larger Pfam alignment, containing 681, 4223 and 788 entries, respectively. Indeed, for these families, Pfam provides both a small and a large MSA; the large MSA contains more distant homologues.

With each of our (native) proteins aligned with an appropriate, Pfam MSA, there is a unique correspondence between positions in the designed sequences and the MSA. We then computed the following similarity score:
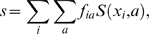
(2)where 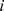 is a position in the designed sequence; 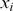 is the amino acid type in the designed sequence at that position; 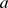 is either one of the 20 amino acid types or a gap symbol; 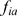 is the frequency (between 0 and 1) of 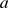 at the corresponding position in the Pfam MSA; and 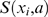 is the BLOSUM62 scoring matrix. Other matrices give similar results [42]. If 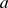 is a gap symbol, 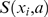 is set to −5. The first sum is over the designed sequence; the second sum is over the amino acid types (including the gap symbol).

### Residual entropy of the natural and designed sequences

To compare the sequence diversity in the designed sequences with the diversity in natural sequences, we used a standard, position-dependent entropy [78], computed as follows:
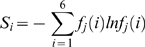
(3)where 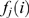 is the frequency of residue type 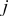 at position 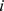, either in the designed sequences or in the natural sequences (organized into an MSA). Instead of the usual, 20 amino acid types, we employ classification systems of either nine or six residue types, corresponding to the following groupings: {LVIMC}, {FY}, {W}, {G}, {A}, {STP}, {EDNQ}, {KR} and {H} (nine groups); or: {LVIMC}, {FYW},{G}, {ASTP}, {EDNQ}, and {KRH}(six groups). This classification is obtained by a cluster analysis of the BLOSUM62 matrix [79], and also by analyzing residue-residue contact energies in proteins [80].

### SUPERFAMILY

SUPERFAMILY [25], [27] is a library of profile Hidden Markov Models [78], designed to associate a protein sequence with the most probable 3D structural model. The library is based on the SCOP classification of proteins, with one model for each protein domain in SCOP. We downloaded the set of models (version 1.69) and used them in connection with the Sequence Alignment and Modeling system (SAM, version 3.5), recommended by the creators of the SUPERFAMILY database. We used our 8,000 lowest-energy sequences as queries against the SUPERFAMILY library; significant hits were returned with the corresponding E-value and domain assignment.

### CDD: a Conserved Domain Database for protein classification

The Conserved Domain Database (CDD) is the protein classification component of NCBI's Entrez query and retrieval system [21]. CDD contains protein domain models imported from outside sources, such as Pfam and SMART, and protein domain models curated at NCBI. In all, CDD contains over 12,000 models. Our designed sequences were queried against the CDD database, run locally. For each redesigned domain we analyzed the 8,000 lowest-energy sequences.

### PSI-BLAST analysis of the designed sequences

For each backbone template, we evaluated the native-like character of the designed sequences using a PSI-BLAST search procedure. We first constructed a PSSM using experimental sequences and one of two different procedures, detailed in the next paragraph. With one of the PSSMs in hand, we then searched a database containing the 8,000 lowest-energy designed sequences along with half of the experimental sequences from the “Non-Redundant” or NR01 database (chosen arbitrarily). By “diluting” the designed sequences within a large set of experimental sequences, we realistically test the ability of PSI-BLAST to identify them. We expect that the exact manner of diluting them is not critical; for example, we could have chosen to add the entire NR01 database instead of half. The database was searched using the program BLASTPGP (running locally).

For the PSI-BLAST analysis just described, and for each backbone template, we used one of two distinct PSSMs. The first is a “general” PSSM, constructed as follows. The native sequence was used to query the NR01 database, through four PSI-BLAST iterations, using a 10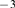 E-value cutoff to define hits. For each backbone template, we were left with about 1000 homologous sequences and a PSSM. The second is a “backbone-specific” PSSM, involving closer homologues: we searched SwissProt with a single PSI-BLAST iteration, collecting about 50 sequences that have at least a 45% identity with the native template, and which define the PSSM.

### Covariance analysis of designed sequences

To characterize correlated mutations within a particular protein family, we use a standard mutual sequence entropy [78], [81]. A correlation coefficient 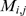 is computed between two amino acid positions 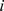, 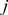 in a given multiple sequence alignment (MSA), for example a collection of designed sequences obtained with a particular backbone template. 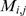 is defined as:
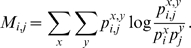
(4)The double sum is over the amino acid types 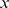 and 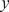, found respectively in columns 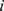 and 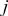 of the MSA; 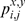 is the joint probability to observe 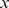 at position 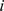 and 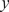 at position 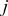; 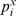 is the probability to observe type 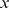 at position 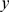; 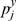 is the probability to observe type 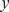 at position 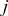. The probabilities are estimated by a simple counting within the MSA columns. If a particular type 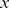 or 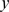, or a pair of types 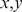 is absent from the corresonding column or pair of columns, the corresponding terms in (4) are set to zero. In (4), we actually use a reduced alphabet of amino acid types, with the following nine types: {LVIMC}, {FY}, {W}, {G}, {A}, {STP}, {EDNQ}, {KR}, and {H}.

## Results

### The designed sequences ressemble experimental sequences

**Figure 1:**
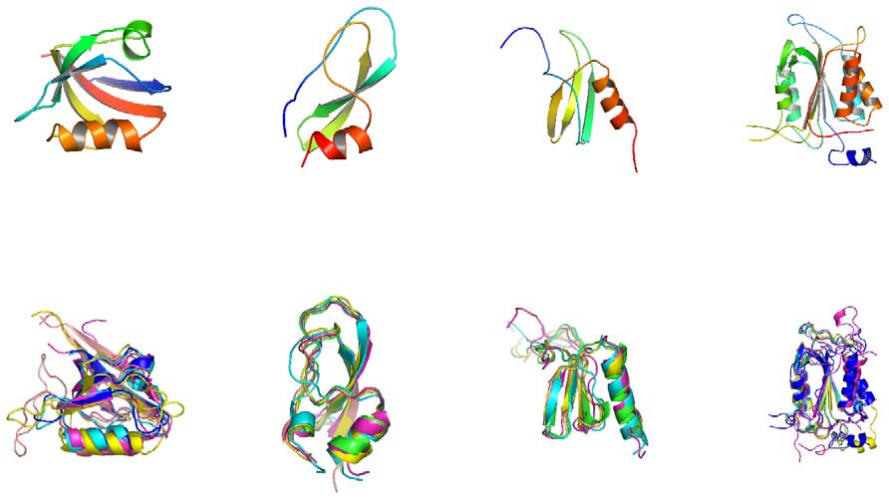
The four SCOP families studied here. From left to right: Small Kunitz-type Inhibitors (SKIs), Chemokines, PDZ domains, and Caspases, represented by a single 3D structure (above) or an alignment of five family members (below).

We redesigned 8 SKIs, 12 Chemokines, 17 PDZ domains and 6 Caspases, listed in Table 1 and illustrated in Fig. 1. Identity rates between the designed sequences and the initial, native sequence are commonly used as a first quality check for CPD, and are given in Table 1. For the 8,000 lowest-energy sequences, the average identity scores are: 40.4% (SKIs), 30.3% (chemokines), 32.3% (PDZ) and 33.8% (caspases), similar to the SH2 and SH3 cases studied earlier [42].

**Table 1:** Identity scores of the low-energy designed sequences^a^.

SKIs		__________ Chemokines __________		PDZ domains		__________ Caspases __________	
PDB code	Identity	PDB code	Identity	PDB code	Identity	PDB code	Identity
4pti	37.4	1ilq	30.3	1pdr	36.0	1nme	31.0
2knt	42.2	1plf	31.3	1kwa	22.8	1m72	31.6
1tfx	41.8	1msg	25.6	2fe5	36.3	1pyo	37.5
1aap	42.6	1hum	32.4	1be9	32.5	1i51	32.1
1bik	42.6	1vmp	32.2	1qav	29.7	1qdu	28.7
1dtx	37.3	1rto	40.1	1nte	24.2	1nw9	41.9
1bun	37.1	1dom	26.0	1l6o	31.7		
1dem	41.9	1eig	27.9	1qau	30.3		
		1g2s	26.5	1g9o	35.4		
		1j9o	18.2	1ihj	28.6		
		1nr2	38.7	1n7f	30.5		
		1nap	30.7	2h3l	36.2		
				1n7e	30.9		
				1q3o	35.6		
				2f5y	35.6		
				2fne	35.2		
				2byg	36.5		
**Mean**	40.4		30.3		32.3		33.8

*^a^*Mean identity of the 8.000 lowest-energy designed sequences relative to each corresponding native template.

Similarity scores are a more reliable measure of the native-like character of designed sequences, because they take into account the diversity of the natural sequences [42], [67]. For each family, we computed the similarity with respect to the small Pfam alignment (see Methods). Table 2 reports the overlap between the similarity scores of the designed sequences and the scores obtained with the natural, Pfam sequences themselves. For the chemokines, only 12% of the designed scores overlap with the scores of the small Pfam set; 73% overlap with the scores of a larger Pfam set (which includes more distant homologues). For the caspases, 24% of the sequences overlap with the small Pfam set; 28% overlap with the large set. For the PDZ domains, 79% overlap with the small Pfam set, and 80% with the large set. For the SKIs, all the designed scores overlap with the scores of the small and large Pfam sets.

**Table 2:** Similarity overlap and recognition rates (%) of designed and random sequences.

	_____________ SKIs _____________				__________ Chemokines __________			
	Designed	R55	R45	R35	Designed	R55	R45	R35
SUPERFAMILY	100	97.9	84.0	53.2	99.7	92.0	66.9	30.1
CDD	100	91.7	62.7	24.1	94.1	90.5	58.8	17.5
PSI-BLAST^a^	95.1	36.1	7.0	0.5	81.0	53.1	11.5	0.8
PSI-BLAST^b^	96.0	87.0	32.7	2.5	88.0	96.2	61.4	7.7
Similarity	99.9	53.2	20.8	0.7	12.1	0.2	0	0
overlap^c^					72.8	21.4	4.0	0.2
FROST	80				81			

	__________ PDZ domains __________				__________ Caspases __________			
	Designed	R55	R45	R35	Designed	R55	R45	R35
SUPERFAMILY	95.4	63.5	21.1	2.3	89.6	98.6	70.8	14.2
CDD	76.4	38.5	10.1	1.4	92.8	99.8	86.6	29.2
PSI-BLAST^a^	80.2	36.8	3.5	0.1	82.5	99.7	68.3	6.9
PSI-BLAST^b^	78.6	99.2	79.2	10.6	92.0	100	99.4	47.4
Similarity	78.6	23.7	2.3	0.1	24.3	11.3	0.5	0.0
overlap^c^					28.5	15.3	0.8	0.0
FROST	64				92			

*^a^*Using the general PSSMs (see Methods).

*^b^*Using the backbone-specific PSSMs.

*^c^*Overlap with the similarity scores for the small (top line) and large (bottom line) Pfam ensembles of natural sequences.

Similarity scores were also computed for random sequences, restrained to have a 35%, 45%, or 55% mean identity with the backbone template. We refer to these as the R35, R45, and R55 sequences. For the SKI and chemokine templates, the random sequences are constrained to maintain the conserved, native cysteines. Results are given in Table 2. The scores of the designed sequences have a much higher overlap with Pfam than the random sequences, even those generated at a 55% identity level (R55 sequences).

### Residual Entropy

**Figure 2:**
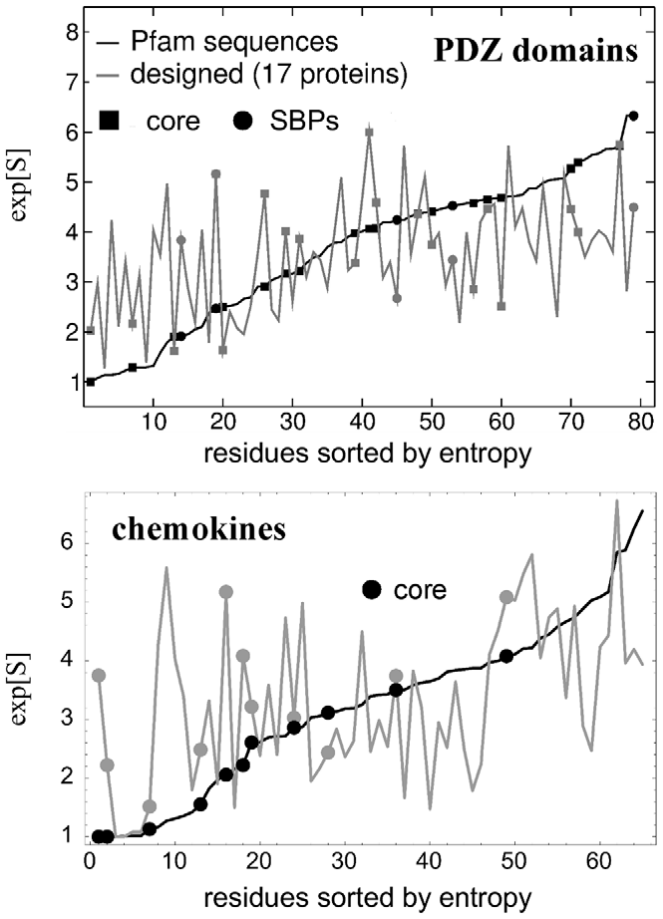
Exponentiated entropy, 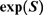, of natural sequences (black line) and designed sequences (grey line). Results computed using a reduced amino acid alphabet with nine classes (see text). Residues are numbered by increasing experimental entropy. Core positions and five Substrate Binding Positions (in the PDZ case; SBPs) are highlighted.

We next consider the diversity of the designed sequence ensembles, using a standard sequence entropy [37], [78]. The 8,000 lowest energy sequences were used. Table 3 gives the (exponentiated) entropy, averaged over the entire polypeptide chain, or over the core positions only (except for the SKIs, where the protein core is very small). Entropies are also given for the natural, Pfam ensembles (the small sets). Agreement between the designed and natural entropies is good, similar to the SH2 and SH3 cases studied earlier [42]. The highest discrepancies are for the SKIs and chemokines, with natural/designed entropies of 3.6/3.0 and 3.5/3.0, respectively. The variation of the (exponentiated) entropy along the polypeptide chain is shown in Fig. 2 for the chemokines and the PDZ domains. The behavior of the designed and natural sequences are qualitatively similar, though the details are different.

**Table 3:** Entropy of natural and designed sequences^a^.

	Amino acids	Pfam sequences^b^	Designed (8 proteins)^b^	Designed (1 protein)^b^
SKIs	All	3.6 (3.0)	3.0 (2.7)	1.8 (1.6)
Chemokines	Core	3.6 (3.1)	3.3 (2.9)	2.2 (1.8)
	All	3.2 (2.8)	3.3 (2.9)	2.0 (1.8)
PDZ domains	Core	3.1 (2.8)	3.6 (3.1)	2.0 (1.8)
	All	3.6 (3.2)	3.5 (3.1)	1.7 (1.6)
Caspases	Core	2.6 (2.3)	2.8 (2.3)	2.5 (2.1)
	All	3.5 (3.0)	3.0 (2.7)	2.1 (1.9)

*^a^*Exponentiated entropies, computed using a simplified amino acid alphabet with nine classes: {LVIMC}, {FY}, {W}, {G}, {A}, {STP}, {EDNQ}, {KR} and {H}, or with six classes (results in parentheses): {LVIMC}, {FYW}, {G}, {ASTP}, {EDNQ}, and {KRH} [80].

*^b^*The corresponding small Pfam set.

*^c^*low-energy sequences from either eight backbone templates or a single template (arbitrarily chosen).

### Fold recognition tools confirm the natural character of designed sequences

The designed sequences were subjected to four standard fold recognition tools: PSI-BLAST, the SUPERFAMILY HMM library, the CDD ressource, and the FROST program [82]. PSI-BLAST was used with several different Position Specific Scoring Matrices (PSSMs; see Methods and [42]). The first, “general” PSSM was constructed from natural sequences from the same family in the NR01 database (a non-redundant subset of SwissProt). For each family, more than 80% of our designed sequences were correctly identified, with E-values below the chosen, 0.001 threshold (Table 2). For the designed SKIs, the detection rate was over 95%.

A second set of 43 “backbone-specific”PSSMs was constructed: one for each designed domain. Each PSSM was constructed using a database of close homologues of the corresponding protein (with at least 45% identity; see Methods). With these PSSMs, the detection rate is higher: 96% for the SKIs, 88% for chemokines, and 92% for the caspases. Only for the PDZ domains, the detection rate is slightly reduced with the backbone-specific PSSMs: 78.6% (instead of 80.2% with the general PSSM). The detection rates compare favorably to those of the random sequences (Table 2).

**Figure 3:**
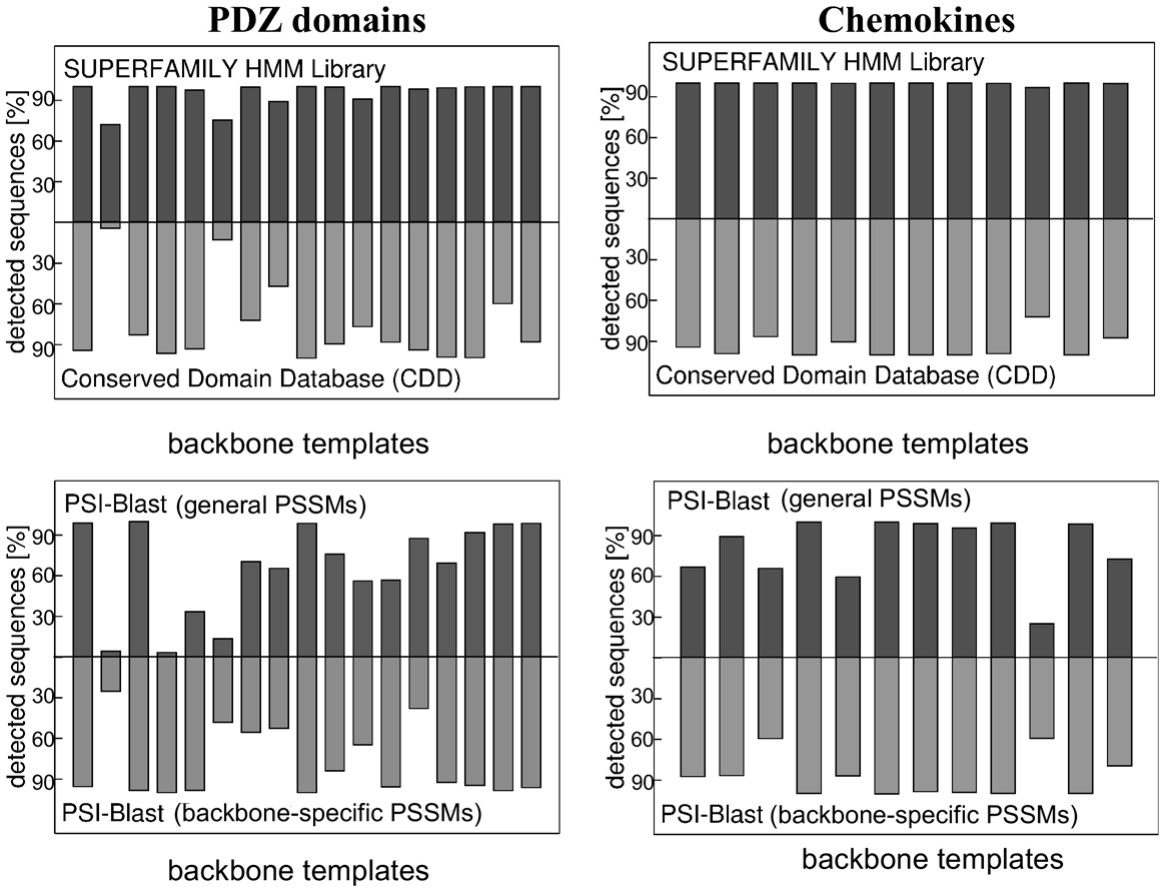
Designed sequences detected as PDZ domains or chemokines by SUPERFAMILY, CDD, and PSI-BLAST. Each column corresponds to one of the backbone templates. For each template, results are shown for the 8,000 lowest-energy sequences.

The SUPERFAMILY HMM library yielded the correct family assignment for the vast majority of designed sequences (Table 2): almost 100% for SKIs and chemokines, and over 90% for caspases and PDZ domains. The 8,000 lowest-energy designed sequences outperformed the random sequences, except for the R55 ones (55% identity to the caspase templates; see Table 2 and Figure 3). The designed caspases outperform the R45 and R35 random sequences.

The CDD library identifies most of the 8,000 lowest-energy designed sequences correctly: 100% of the SKIs, 94% of the chemokines, 76% of the PDZ domains, and 93% of the caspases (see Table 2 and Figure 3). For three families, the detection rates of the designed sequences exceed those of the random sequences; only the caspases perform less well than the R55 random sequences (as with SUPERFAMILY).

Finally, the designed sequences were evaluated by the FROST library of threading models [42], [82]. For each backbone template, we evaluated around 200 low-energy designed sequences, chosen randomly. About 20% of the 1600 SKI and 2400 chemokine sequences were assigned to an incorrect family or not assigned at all by FROST; the other 80% were assigned to the correct family (Table 2). 64% of the PDZ sequences were correctly assigned, and 92% of the caspase sequences. Overall, the designed sequences have a good native-like character, much stronger than the R55 random sequences. The precise rate of detection varies somewhat between PSI-BLAST, SUPERFAMILY, CDD, and FROST.

### Stability of the designed and native sequences; relation to sequence identity

**Figure 4:**
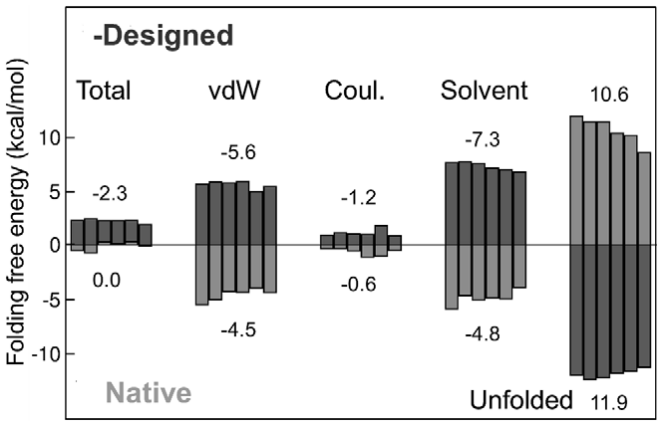
Individual components of the folding free energy 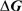, on a per-residue basis. Results are for six protein templates (the six bars that appear for each energy term). From left to right: 1CKA and 1CSK (SH3 domains); 1NRV and 1SHD (SH2 domains); 1QAU and 2FE5 (PDZ domains). Dark bars correspond to the 8,000 lowest-energy designed sequences; light bars correspond to native sequences with optimized rotamers. Mean values (kcal/mol) are given above or below each set of columns. The designed and native sequences use opposite sign conventions, for clarity (as if we plotted the negative designed energies).

To further understand the energy terms that drive the design, we performed a component analysis of the folding free energy, 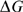. We distinguished the four terms in the CASA energy function (see Methods): the van der Waals, screened Coulomb, and surface area terms in the folded state (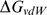, 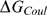, 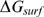), and the unfolded state energy (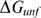). Results were normalized by the protein chain length, yielding mean residue contributions. The analysis was done for two PDZ domains, as well as two SH2 and SH3 domains, studied earlier [42]. For the designed sequences, on average, each residue contributes −2.3
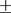
0.2 kcal/mol to 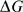 (Fig. 4). For all six proteins, 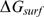 is the largest folded state component (−7.3
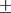
0.3 kcal/mol), followed by 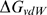 (−5.6
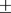
0.3 kcal/mol), and 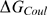 (−1.2
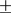
0.3 kcal/mol). The negative sign indicates contributions that favor folding. The mean unfolded state contribution is 11.9
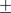
0.4 kcal/mol for the designed sequences. The positive sign indicates an unfavorable contribution to folding.

For the native sequences, after optimizing the sidechain rotamers (for consistency with the designed sequences), the stability is weaker, with a mean folding free energy of 0.0
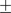
0.4 kcal/mol (per residue). Compared to the designed sequences, the surface and van der Waals terms are less favorable with the native sequences; this is only partly compensated for by a less stable unfolded state for the native sequences (10.6 kcal/mol per residues, vs. 11.9 kcal/mol for the designed sequences; Fig. 4). The designed sequences are thus overstabilized, probably because of our optimization procedure, which maximizes stability. Real proteins are obviously subject to other selective pressures, including functional pressure.

**Figure 5:**
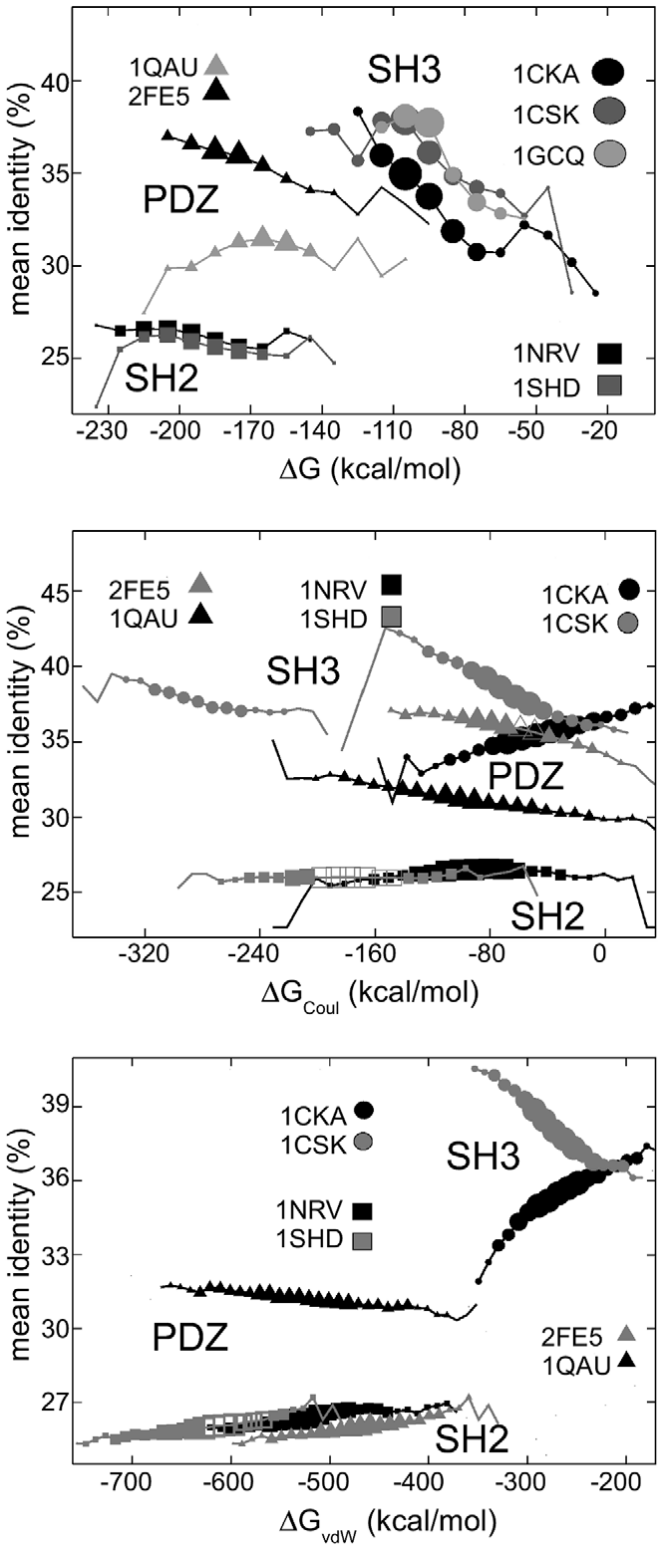
Mean identity score *vs.* the folding free energy 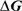 (top) and its components (middle, bottom), for seven proteins. Results are for the 8,000 lowest-energy designed sequences, which are compared to their corresponding native template. The size of each symbol indicates the number of sequences with the corresponding energy (energies binned in 10 kcal/mol windows). Negative energies indicate stable folding of the designed sequences.

The enhanced stability of the designed sequences prompted us to compare sequence “quality” to protein stability. Specifically, Fig. 5 shows the sequence identity of the 8,000 lowest-energy designed sequences (relative to the native template) as a function of the computed folding free energy, 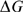. In five out of six cases, the identity scores of the designed sequences improve as 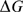 improves; i.e., the lowest-energy designed sequences have the best identity score. The SH3 graphs are clearly separated from the others, with a more negative slope. The best SH3 sequences are 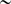
100 kcal/mol below the highest 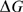 value. For the two SH2 proteins, the curves are flatter, but there is still a slight increase in the identity scores as 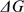 improves. For the 2FE5 PDZ domain, the identity scores of the designed sequences also increase as 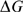 improves. Only for 1QAU, the identity score does not improve with 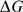, and actually gets worse for the most stable sequences. This provides support for using the folding free energy as a selection criterion, despite the overstabilization seen in Fig. 4.

Fig. 5 also shows the relation between the sequence identity and the individual, van der Waals and screened Coulomb components of 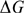. Again, results are for the 8,000 lowest-energy designed sequences, compared to the corresponding native template. In some cases, each component improves along with the identity (1CSK, 1QAU); in others, only one or the other component improves along with the identity. For 1CKA, it is the solvation component that improves with the identity.

### Homologue searching using designed sequences and PSSMs

Our longer-term goal is to use designed sequences for homologue detection, in combination with natural sequences [40]. Following our previous study [42], we constructed “theoretical” PSSMs from the designed sequences and used them for homologue searching. In the chemokine case, for comparison, we also constructed a PSSM from the most “native-like” designed sequences: those that gave the lowest E-values for the CDD calculations described above. For the PDZ family, we also considered the effect of resetting a few functional positions to their native amino acid types. Specifically, we identified five substrate-binding positions, or SBPs from a literature search [83], .

We compared the performance of the different “designed” PSSMs to experimental PSSMs, constructed using the same procedure, with the NR01 database replacing the ensemble of designed sequences. Random PSSMs were also employed, with pools of 1000 random sequences replacing the designed or NR01 ensembles [42]. The identity levels for the random sequences were 35%, 45%, or 55%, as before; we refer to them again as the R35, R45, and R55 sequences. We use an E-value threshold of 0.1 for sequence retrieval [42].

**Figure 6:**
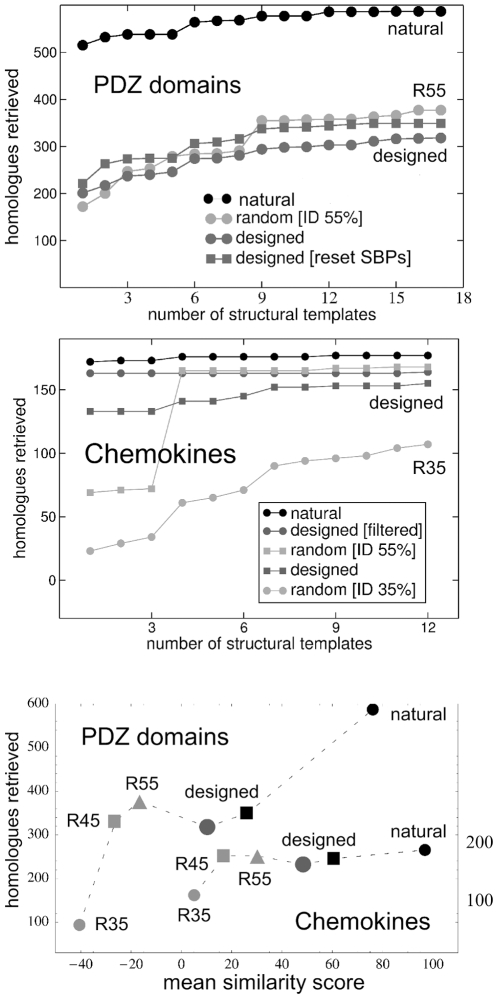
Homologues retrieved from Swissprot using natural, designed, and random sequences. PDZ domains (top) and chemokines (middle); cumulative number retrieved as more templates are considered. Selected curves are labelled, for clarity. For the PDZ domains (upper panel), the grey squares correspond to designed sequences with five Substrate Binding Positions reset to their experimental amino acid types. Bottom panel: retrieval rate *vs.* the mean similarity scores of the sequences employed (with respect to the Pfam sequences). Chemokine results correspond to the righthand axis.

Results are summarized in Table 4 and Fig. 6. The best results are for the STIs and the chemokines. The experimental STI PSSMs retrieve 129 STIs from Swissprot, compared to 123 with the designed PSSMs, 128 with the R55 sequences, 126 with R45 and 71 with R35. The random PSSMs give several false positives; the designed PSSMs give none. The different PSSMs compare similarly when the search is performed within the PDB database (not shown). For the chemokines, the experimental PSSMs retrieve 177 sequences; the designed sequences, 155. With the most “native-like” designed sequences, we retrieve 164 of the 177 (93%). Finally, the R55 and R45 sequences retrieve more sequences (168 out of 177), but give more false positives (Table 4). There is a large jump in the R55 curve, between the 3rd and 4th backbone templates. This occurs because template 4 belongs to the CC subclass within the chemokine family, whereas templates 1–6 belong to the second, CXC subclass. These subclasses differ by the positioning of two cysteine residues; since the cysteines are not randomized, the R55 sequence behavior is different depending on the subclass of the native template. The effect of the cysteines on the rate of retrieval with the designed sequences is much smaller.

**Table 4:** Swissprot sequences retrieved using natural, designed, and random PSSMs.

PSSM^a^	SKIs	Chemokines	PDZ domains	Caspases
Natural	129 (0)	177 (1)	587 (5)	75 (2)
Designed	123 (0)	155 (2)	318 (10)	40 (3)
Designed^b^		164 (0)	350 (12)	
R55	128 (8)	168 (6)	377 (11)	62 (20)
R45	126 (3)	168 (6)	331 (38)	59 (25)
R35	71 (4)	107 (8)	94 (41)	33 (15)

Number of false positives in parantheses.

*^a^*The sequences used to construct the PSSM are either natural sequences from the NR01 database, low-energy designed sequences, or random sequences.

*^b^*The designed sequences with the highest CDD scores (Chemokines) or with five SBPs reset to their native types (PDZ domains).

For the PDZ and caspase families, the retrieval rates are much lower: 54% and 53% of the experimental hits are retrieved (compared to 95% and 88% for the STIs and chemokines). If the five SBPs are reset to their experimental amino acid types, the PDZ rate improves to 60% (350 correct hits, vs. 587 with the experimental PSSMs). The performance is greater than with the R35 sequences, but somewhat less than with the R45 ones. Overall, the PDZ and caspase results are somewhat poorer than for the earlier SH2 and SH3 cases, while the STI and chemokine results are far better. Evidently, the ability to retrieve homologues depends on the fold, with the conserved cysteine pattern in the STIs and chemokines probably playing a role. The chemokine results would improve further if we considered more SCOP templates (in addition to the 12 used here).

### Restrained sequence optimization shows that amino acid positions are correlated

The limited ability of the designed PDZ and caspase sequences to retrieve homologues contrasts with the ability of the SUPERFAMILY HMMs to recognize them as native-like. This may indicate that too much sequence information is lost when PSI-BLAST is used for homologue searching, since PSI-BLAST replaces the designed sequences by a profile. In the profile, correlations between amino acid positions are averaged out. A full correlation analysis is beyond the scope of this article and will be reported elsewhere. However, in this section and the next, we provide evidence that such correlations are important in the designed sequences. We first compare the designed sequences to ones produced by a SUPERFAMILY HMM, or “HMM sequences”. The HMM generates random sequences that obey the (position-dependent) amino acid probabilities in the experimental sequences, but not the correlations between positions. We next compare to sequences obtained through a restrained optimization, where correlations can partially develop. Both the HMM sequences and the restrained optimization lead to structures that cannot pack in a stable manner, and have unfavorable values of the folding free energy, 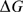. We considered two PDZ domains, two SH2 domains, and two SH3 domains, as in the stability analysis, above.

**Figure 7:**
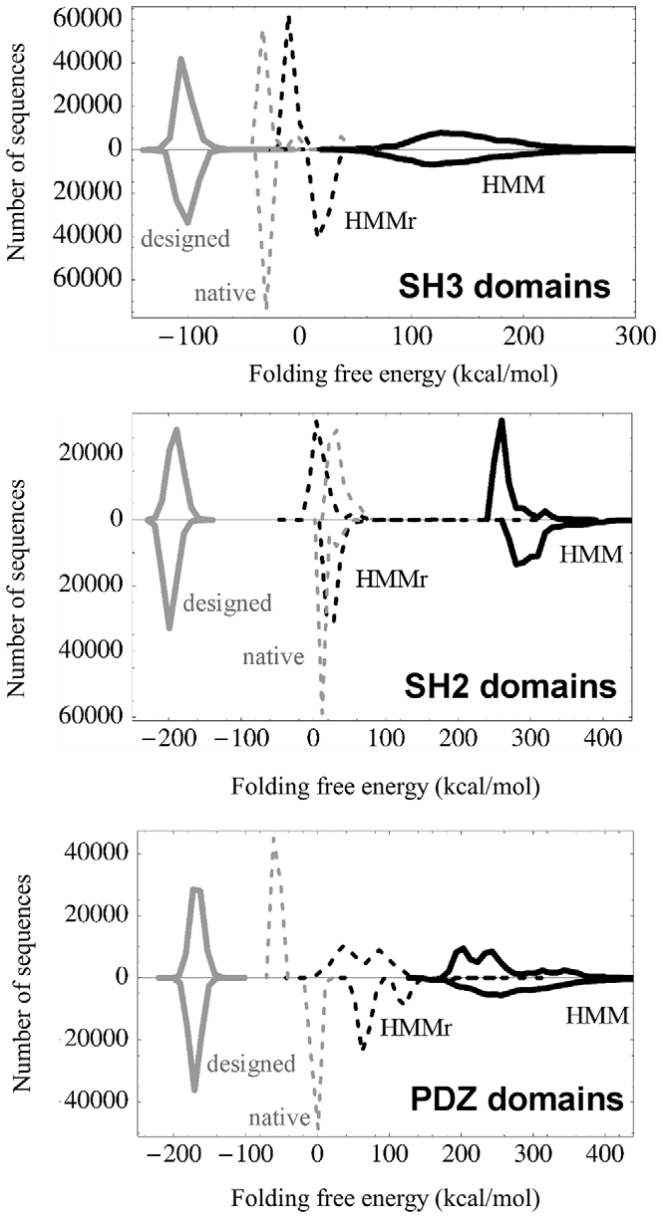
Histograms of the folding free energy, 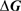. Results are shown for designed, native and HMM sequences, for two SH3 domains (1CKA, 1CSK), two SH2 domains (1SHD,1NRV), and two PDZ domains (1QAU, 2FE5). Black: HMM; grey: designed; dashed grey: native; dashed black: HMM sequences after restrained optimization (using 9 amino acid groups). Each panel shows data for two proteins, with opposite vertical axes.

Results are summarized in Figure 7, and are similar for all six proteins. The designed sequences, which are fully optimized in both sequence and rotamer space, have large, negative folding free energies. The HMM sequences, in contrast, have very unfavorable, positive folding free energies. The HMM sequences are drawn from the experimental profile and subjected to rotamer optimization but not sequence optimization. Their poor stability suggests that when sequences do not respect the interactions and correlations between amino acids, they are unable to pack in a stable way.

In a second step, we allow the HMM sequences to partially optimize. We define a reduced alphabet of nine amino acid groups, or “flavors”: {LVIMC}, {FY}, {W}, {G}, {A}, {STP}, {EDNQ}, {KR}, and {H} (see Methods). In the restrained optimization, each amino acid is allowed to vary its type, but not its flavor; *e.g.*, a Phe can mutate into Tyr but not into Trp. The restrained optimization improves the folding free energies considerably, so that the optimized values are in between the HMM and designed values. This indicates that correlated mutations have a large effect on the stability. Importantly, unrestrained optimization of the HMM sequences gives an energy spectrum that is indistinguishable from the designed spectrum (not shown).

For completeness, we also analyzed the native sequences (with optimized rotamers). We see that the designed sequences are overstabilized, compared to the native sequences (Figure 7), as already noted.

### Analysis of the correlated mutations in a PDZ domain

**Figure 8:**
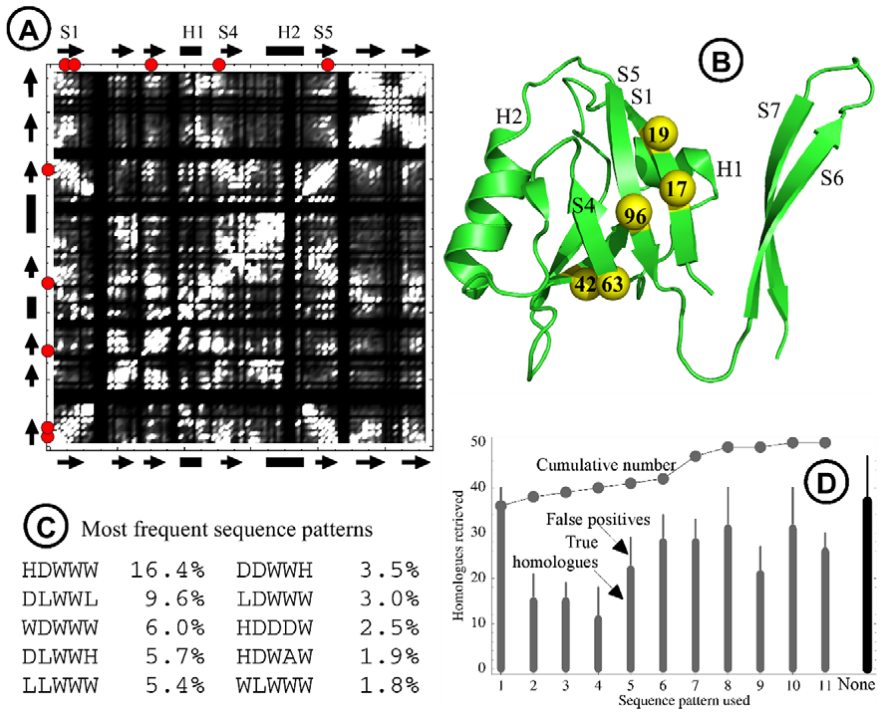
Correlation analysis for the PDZ domain 1QAU. **A**) Covariance matrix; the amino acid sequence runs along the top and the side of the plot, with secondary structure elements indicated as arrows (strands) or rectangles (helices). Bright points in the matrix correspond to higly-correlated amino acid pairs. Red dots along the top and side label the network shown in B). **B**) 3D structure with secondary structure elements labelled as in A). A correlated network of five amino acids is shown (yellow spheres, labelled with amino acid number; red dots in A). **C**) The most frequent sequence patterns for the five amino acids, with their frequency within the 10,000 low energy sequences. **D**) Number of homologues retrieved by BLAST searching using subsets of sequences that obey one of the frequent patterns (E-value threshold of 1). Homologues retrieved using all the low energy sequences are shown by the rightmost bar (labelled ‘None’). Thick lines represent true homologues; thin lines show false positives.

To further characterize the correlations between amino acid positions, we consider a single example, the PDZ domain 1QAU. For this protein, we have analyzed the correlated mutations that occur within the set of 10,000 (not 8,000) low-energy designed sequences. A correlation coefficient was defined in Methods, which is effectively a mutual sequence entropy [78], [81]. Considering all pairs of positions in 1QAU, we obtain a covariance matrix, which is shown in Fig. 8A. By inspecting the matrix and the protein 3D structure, we identified a small network of five amino acids that are strongly correlated; Fig. 8B shows them in the context of the 3D structure. The different amino acid sequences that occur most frequently at these five positions, within the set of designed sequences, are shown in Fig. 8C. In fact, the sequences are described with the reduced alphabet of nine ‘flavors’ defined in the previous section. Therefore, we speak of ‘sequence patterns’, rather than sequences. The two most frequent sequence patterns are HDWWW (16.4% of the 10,000 low-energy sequences, or 1640 sequences) and DLWWL (9.6% of the sequences).

If the observed correlations are realistic, we may expect that similar sequence patterns should be present in the experimental PDZ sequences. For example, subsets of the experimental sequences would be distinctly more similar to one of the Fig. 8C patterns than to another. A detailed and comprehensive test of this hypothesis will be difficult and is left for future work. However, a preliminary test is reported here. The test consists in using the different sequence patterns (Fig. 8C) individually to do homologue searching. For a given pattern, say HDWWW, we first isolate the subset of designed sequences that contain this pattern (1640 sequences). From these sequences, we randomly select 50 sequences and query NR01 with the corresponding profile. This procedure is repeated ten times for the given subset and the retrieved homologues (with an E-value threshold of 1) are recorded. Fig. 8D summarizes the results obtained with the ten most frequent sequence patterns. The procedure is also done using the designed sequences that do not contain any of the ten patterns (4420 sequences, forming an 11th subset). The number of homologues retrieved using each pattern is shown, as well as the total number, cumulated over all patterns. For comparison, we did the same thing with the entire pool of 10,000 designed sequences, instead of the subsets. To make the comparison “fair”, we did 110 repetitions when all the sequences are pooled (compared to 10 repetitions for each of the 11 subsets). With the individual patterns and subsets of sequences, we retrieve between 11 and 36 homologues, depending on the pattern, and 4–9 false positives. When we cumulate over all subsets (including the 11th subset, made of the sequences that do not obey any of the top ten patterns), we obtain 50 homologues (and 22 false positives), compared to 37 (and 10) when the entire pool of sequences is used directly. If the procedure is repeated with an E-value threshold of 0.01, we obtain a total of 8 homologues using the sequence patterns (plus one false positive), *versus* 6 using the entire pool of sequences (plus one false positive). Thus, the sequence pattern analysis allows us to identify several new homologues of 1QAU, suggesting that additional information can be retrieved if the correlations in the designed sequences are exploited. In the future, we will apply this analysis more systematically.

## Discussion

We have applied a design method to 43 proteins, belonging to four SCOP families, extending and generalizing our previous study of 46 SH2 and SH3 domains [42]. The four families present different challenges. The proteins are larger than the SH3 and SH2 domains. For the small SKI and caspase families, we designed essentially the entire SCOP sets. For the larger PDZ and chemokine families, we designed, respectively, 1/3 and 1/2 of the Xray structures available in SCOP. Natural PDZ domains, in spite of their structural similarity, have a low, average, mutual sequence identity of just 24%. 16 distinct specificity classes were recently reported for the PDZ domain family [84]. The classes are defined by just a few amino acids, which are fine-tuned to interact with specific protein partners. In some of our calculations, five of these positions (SBPs) were reset to their native types. The SKIs and chemokines, on the other hand, each contain a conserved network of cysteines, which form a distinctive fingerprint.

Overall, the identity scores reported here are comparable to our previous results for SH2 and SH3 domains [42], [67], [68] and to those of other groups [35]–[39], [47]–[50], [52]–[65]. SUPERFAMILY, CDD and FROST results agree with our previous study, where detection rates for designed sequences were 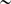
80%. The PSI-Blast detection rates found here are improved (over 80%), especially compared to the designed SH3 sequences (around 50%). The sequence entropies are comparable to those of the natural sequence ensembles, as long as we take into account the full set of backbone templates. If designed sequences from a single template are used, the entropies are too low. By using many representatives of each SCOP family, we introduce backbone variations that are lost at the level of each individual template because of the fixed backbone approximation.

The performance of the designed sequences for homologue detection was investigated by PSI-Blast searching in SwissProt. Designed SKI PSSMs retrieved 95% of the experimental homologues; designed chemokine PSSMs retrieved 88%; the best designed chemokine sequences retrieved 93%. The mean identity of the designed sequences to their respective templates is 40% for the SKIs and 30% for the chemokines. For homologue retrieval, the designed sequences behave like random sequences of 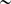
50% sequence identity. This good performance is partly due to the cysteine patterns, which serve as a fingerprint for both families. It is distinctly better than our earlier result for the SH2 family [42], which belongs to the same structural class (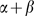) as the chemokines; this suggests that the effects of the structural class are complex. The designed PDZ domains and caspases give poorer PSSMs, with homologue retrieval rates of 53–54%. One limitation of the designed sequences is that they do not include explicit selection for function. On the contrary, by selecting for stability, we discourage some functional mutations, since functional positions are often thermodynamically destabilizing [85], [86]. When just five substrate binding positions (SBPs) in the PDZ domains are reset to their native types, the PDZ homologue retrieval rate increases from 54% to 60%.

Another limitation concerns the PSI-BLAST detection method itself, rather than the designed sequences. Like many fold recognition tools, PSI-BLAST relies on profiles, thereby replacing the ensemble of designed sequences by a single, mean sequence. This averaging eliminates information on correlated mutations within each protein structure. A full analysis of these correlations and their effect on homologue retrieval is beyond the scope of this article, and will be reported elsewhere. However, the folding energy analysis reported here shows that 3D correlations have a large effect on the designed sequences, as expected, and as shown experimentally for designed WW domains [70]. In particular, sequences that obey the experimental, position-dependent, amino acid probabilities, but not the correlations (“HMM sequences”; Fig. 7), have terrible folding free energies. Restrained optimization, where the amino acid types can only change within small groups (“flavors”) gives only a partial improvement, showing that significant changes in the sidechain physical chemistry are needed before the HMM sequences can pack. It remains to be seen whether the correlations can provide a useful additional signal for database searching. A detailed analysis of the correlation patterns, illustrated above for 1QAU, could help extract more information from the designed sequences and might lead to improved homologue retrieval, but this remains to be tested.

Despite the limitations discussed, homologue retrieval for two families is excellent, out of the six families studied so far. With further improvements, and in combination with experimental sequences, CPD could develop into a useful aid for homologue retrieval and fold recognition.

## References

[1] Margulies M, Egholm M, Altman WE, Attiya S, Bader JS (2005). Genome sequencing in microfabricated high-density picolitre reactors.. Nature.

[2] Todd AE, Marsden RL, Thornton JM, Orengo CA (2005). Progress of structural genomics initiatives: An analysis of solved target structures.. J Mol Biol.

[3] George RA, Spriggs RV, Bartlett GJ, Gutteridge A, MacArthur MW (2005). Effective function annotation through catalytic residue conservation.. Proc Natl Acad Sci USA.

[4] Sillitoe I, Dibley M, Bray J, Addou S, Orengo C (2005). Assessing strategies for improved superfamily recognition.. Prot Sci.

[5] Lee D, Redfern O, Orengo C (2007). Predicting protein function from sequence and structure.. Nature Rev Molec Cell Biol.

[6] Liolios K, Tavernarakis N, Hugenholtz P, Kyrpides NC (2006). The genomes on line database (GOLD) v.2: a monitor of genome projects worldwide.. Nucl Acids Res.

[7] Benson DA, Karsch-Mizrachi I, Lipman DJ, Ostell J, Wheeler L (2006). GenBank.. Nucl Acids Res.

[8] Dessailly B, Nair R, Jaroszewski L, Fajardo JE, Kouranov A (2009). PSI-2: Structural genomics to cover protein domain family space.. Structure.

[9] Chandonia JM, Brenner SE (2006). The impact of structural genomics: Expectations and outcomes.. Science.

[10] Levitt M (2009). Nature of the protein universe.. Proc Natl Acad Sci USA.

[11] Andreeva A, Howorth D, Brenner SE, Hubbard JJ, Chothia C (2004). SCOP database in 2004: refinements integrate structure and sequence family data.. Nucl Acids Res.

[12] Andreeva A, Howorth D, Chandonia JM, Brenner SE, Hubbard TJP (2008). Data growth and its impact on the SCOP database: new developments.. Nucl Acids Res.

[13] Pearl F, Todd A, Sillitoe I, Dibley M, Redfern O (2005). The CATH domain structure database and related resources Gene3D and DHS provide comprehensive domain family information for genome analysis.. Nucl Acids Res.

[14] Berman HM, Westbrook J, Feng Z, Gilliland G, Bhat TN (2000). The Protein Data Bank.. Nucl Acids Res.

[15] Marti-Renom MA, Stuart AC, Fiser A, Sanchez R, Melo F (2000). Comparative protein structure modeling of genes and genomes.. Annu Rev Biophys Biomol Struct.

[16] Venclovas C (2001). Comparative modeling of CASP4 target proteins: Combining results of sequence search with three-dimensional structure assessment.. Proteins.

[17] Schwede T, Kopp J, Guex N, Peitsch MC (2003). Swiss-Model: an automated protein homology-modeling server.. Nucl Acids Res.

[18] Altschul SF, Madden TL, Schaffer AA, Zhang JH, Zang Z (1997). Gapped BLAST and PSI-BLAST: a new generation of protein database search programs.. Nucl Acids Res.

[19] Schaffer AA, Aravind L, Madden TL, Shavirin JL, Spouge S (2001). Improving the accuracy of PSI-BLAST protein database searches with composition-based statistics and other refinements.. Nucl Acids Res.

[20] Karplus K, Barrett C, Cline M, Diekhans M, Grate L (1999). Predicting protein structure using only sequence information.. Proteins.

[21] Marchler-Bauer A, Anderson JB, Cherukuri PF, DeWweese-Scott C, Geer LY (2005). CDD: a conserved domain database for protein classification.. Nucl Acids Res.

[22] Bateman A, Birney E, Durbin R, Eddy SR, Finn RD (1999). Pfam 3.1: 1313 multiple alignments and profile HMMs match the majority of proteins.. Nucl Acids Res.

[23] Bateman A, Coin L, Durbin R, Finn RD, Hollich V (2004). The Pfam protein families database.. Nucl Acids Res.

[24] Finn RD, Tate J, Mistry J, Coggill PC, Sammut SJ (2008). The Pfam protein families database.. Nucl Acids Res.

[25] Gough J, Karplus K, Hughey R, Chothia C (2001). Assignment of homology to genome sequences using a library of hidden Markov models that represent all proteins of known structure.. J Mol Biol.

[26] Madera M, Gough J (2002). A comparison of profile hidden Markov model procedures for remote homology detection.. Nucl Acids Res.

[27] Madera M, Vogel C, Kummerfeld SK, Chothia C, Gough J (2004). The SUPERFAMILY database in 2004: additions and improvements.. Nucl Acids Res.

[28] Krogh A, Brown M, Mian IS, Sjolander K, Haussler D (1994). Hidden Markov models in computational biology: applications to protein modelling.. J Mol Biol.

[29] Finn RD, Mistry J, Schuster-Böckler B, Griffiths-Jones S, Hollich V (2006). Pfam: clans, web tools and services.. Nucl Acids Res.

[30] Sonnhammer EL, Eddy SR, Birney E, Bateman A, Durbin R (1998). Pfam: multiple sequence alignments and HMM-profiles of protein domains.. Nucl Acids Res.

[31] Wang Y, Sadreyev RI, Grishin NV (2009). PROCAIN: protein profile comparison with assisting information.. Nucl Acids Res.

[32] Liu X, Fang K, Wang W (2004). The number of protein folds and the distribution over families in nature.. Proteins.

[33] Guerler A, Knapp EW (2008). Novel protein folds and their nonsequential structural analogs.. Prot Sci.

[34] Koehl P, Levitt M (1999). De novo protein design. II. Plasticity in sequence space.. J Mol Biol.

[35] Larson S, Garg A, Desjarlais J, Pande V (2003). Increased detection of structural templates using alignments of designed sequences.. Proteins.

[36] Larson S, Pande V (2003). Sequence optimization for native stability determines the evolution and folding kinetics of a small protein.. J Mol Biol.

[37] Larson S, England JE, Desjarlais J, Pande V (2002). Thoroughly sampling sequence space: Large-scale protein design of structural ensembles.. Prot Sci.

[38] Dantas G, Kuhlman B, Callender D, Wong M, Baker D (2003). A large test of computational protein design: Folding and stability of nine completely redesigned globular proteins.. J Mol Biol.

[39] Saunders C, Baker D (2005). Recapitulation of protein family divergence using flexible backbone protein design.. J Mol Biol.

[40] Zhou H, Zhou Y (2005). Fold recognition by combining sequence profiles derived from evolution and from depth-dependent structural alignment of fragments.. Proteins.

[41] Ding F, Dokholyan NV (2006). Emergence of protein fold families through rational design.. PLOS Comp Biol.

[42] Schmidt am Busch M, Mignon D, Simonson T (2009). Computational protein design as a tool for fold recognition.. Proteins.

[43] Ponder J, Richards FM (1988). Tertiary templates for proteins: Use of packing criteria in the enumeration of allowed sequences for different structural classes.. J Mol Biol.

[44] Hellinga H, Richards F (1994). Optimal sequence selection in proteins of known structure by simulated evolution.. Proc Natl Acad Sci USA.

[45] Dahiyat BI, Mayo SL (1996). Protein design automation.. Prot Sci.

[46] Harbury PB, Plecs JJ, Tidor B, Alber T, Kim PS (1998). High-resolution protein design with backbone freedom.. Science.

[47] Dokholyan NV, Shakhnovich EI (2001). Understanding hierachical protein evolution from first principles.. J Mol Biol.

[48] Desjarlais J, Handel T (1999). Sidechain and backbone flexibility in protein core design.. J Mol Biol.

[49] Kuhlman B, Baker D (2000). Native protein sequences are close to optimal for their structures.. Proc Natl Acad Sci USA.

[50] Wernisch L, Héry S, Wodak S (2000). Automatic protein design with all atom force fields by exact and heuristic optimization.. J Mol Biol.

[51] Jaramillo A, Wernisch L, Héry S, Wodak S (2002). Folding free energy function selects native-like protein sequences in the core but not on the surface.. Proc Natl Acad Sci USA.

[52] Kuhlman B, Dantas G, Ireton G, Varani G, Stoddard B (2003). Design of a novel globular protein fold with atomic-level accuracy.. Science.

[53] Dwyer M, Looger L, Hellinga H (2004). Computational design of a biologically active enzyme.. Science.

[54] Havranek J, Harbury P (2003). Automated design of specifity in molecular recognition.. Nat Struct Biol.

[55] Ventura S, Serrano L (2004). Designing proteins inside out.. Proteins.

[56] Wollacott AM, Zanghellini A, Murphy P, Baker D (2007). Prediction of structures of multidomain proteins from structures of the individual domains.. Prot Sci.

[57] Swift J, Wehbi WA, Kelly BD, Stowell XF, Saven JG (2006). Design of functional ferritin-like proteins with hydrophobic cavities.. J Am Chem Soc.

[58] Kang SG, Saven JG (2007). Computational protein design: structure, function and combinatorial diversity.. Curr Opin Chem Biol.

[59] Koehl P, Levitt M (1999). De novo protein design. I. In search of stability and specificity.. J Mol Biol.

[60] Koehl P, Levitt M (1999). Structure-based conformational preferences of amino acids.. Proc Natl Acad Sci USA.

[61] Hubner IA, Deeds EJ, Shakhnovich EI (2006). Understanding ensemble protein folding at atomic detail.. Proc Natl Acad Sci USA.

[62] Pokala N, Handel T (2004). Energy functions for protein design I: Efficient and accurate continuum electrostatics and solvation.. Prot Sci.

[63] Pokala N, Handel TM (2005). Energy functions for protein design: Adjustement with protein-protein complex affinities, models for the unfolded state, and negative design of solubility and specificity.. J Mol Biol.

[64] Chowdry AB, Reynolds KA, Hanes MS, Voorhies M, Pokala N (2007). An object-oriented library for computational protein design.. J Comp Chem.

[65] Raha K, Wollacott AM, Italia MJ, Desjarlais JR (2000). Prediction of amino acid sequence from structure.. Prot Sci.

[66] Lopes A, Aleksandrov A, Bathelt C, Archontis G, Simonson T (2007). Computational sidechain placement and protein mutagenesis with implicit solvent models.. Proteins.

[67] Schmidt am Busch M, Lopes A, Mignon D, Simonson T (2008). Computational protein design: software implementation, parameter optimization, and performance of a simple model.. J Comp Chem.

[68] Schmidt am Busch M, Lopes A, Amara N, Bathelt C, Simonson T (2008). Testing the coulomb/accessible surface area solvent model for protein stability, ligand binding, and protein design.. BMC Bioinformatics.

[69] Panchenko AR, Bryant SH (2002). A comparison of position-specific score matrices based on sequence and structure alignments.. Prot Sci.

[70] Socolich M, Lockless SW, Russ WP, Lee H, Gardner KH (2005). Evolutionary information for specifying a protein fold.. Nature.

[71] Wlodawer A, Deisenhofer J, Huber R (1987). Comparison of two highly refined structures of bovine pancreatic trypsin inhibitor.. J Mol Biol.

[72] Tuffery P, Etchebest C, Hazout S, Lavery R (1991). A new approach to the rapid determination of protein side chain conformations.. J Biomol Struct Dyn.

[73] Brooks B, Bruccoleri R, Olafson B, States D, Swaminathan S (1983). Charmm: a program for macromolecular energy, minimization, and molecular dynamics calculations.. J Comp Chem.

[74] Lee B, Richards F (1971). The interpretation of protein structures: estimation of static accessibility.. J Mol Biol.

[75] Street A, Mayo S (1998). Pairwise calculation of protein solvent-accessible surface areas.. Folding and Design.

[76] Brünger AT (1992). X-plor version 3.1, A System for X-ray crystallography and NMR.

[77] Anderson DP (2004). BOINC: A system for public-resource computing and storage.. 5th IEEE/ACM International Workshop on Grid Computing.

[78] Durbin R, Eddy SR, Krogh A, Mitchison G (2002). Biological sequence analysis.

[79] Murphy LR, Wallqvist A, Levy RM (2000). Simplified amino acid alphabets for protein fold recognition and implications for folding.. Prot Eng.

[80] Launay G, Mendez R, Wodak SJ, Simonson T (2007). Recognizing protein-protein interfaces with empirical potentials and reduced amino acid alphabets.. BMC Bioinf.

[81] Halperin I, Wolfson H, Nussinov R (2006). Correlated mutations: Advances and limitations. A study on fusion proteins and on the cohesion-dockerin families.. Proteins.

[82] Marin A, Pothier J, Zimmermann K, Gibrat JF (2002). FROST: a filter-based fold recognition method.. Proteins.

[83] Lin D, Gish GD, Songyang Z, Pawson T (1999). The carboxyl terminus of B class ephrins constitutes a PDZ domain binding motif.. J Biol Chem.

[84] Tonikian R, Zhang YN, Sazinsky SL, Currell B, Yeh JH (2008). A specifity map for the PDZ domain family.. Plos Biology.

[85] Shoichet BK, Baase WA, Kuroki R, Matthews BW (1995). A relationship between protein stability and protein function.. Proc Natl Acad Sci USA.

[86] Elcock AH (2001). Prediction of functionally important residues based solely on the computed energetics of protein structure.. J Mol Biol.

